# Living with the aftermath: family experiences of managing concussion and sport-related head injury

**DOI:** 10.3389/fspor.2026.1867508

**Published:** 2026-08-13

**Authors:** Ed Daly, Lisa Ryan

**Affiliations:** Department of Sport, Exercise & Nutrition, School of Health, Sport Science & Nutrition, Atlantic Technological University - Galway City, Galway, Ireland

**Keywords:** chronic traumatic encephalopathy, concussion, family experiences, moral injury, neurodegeneration, reflexive thematic analysis, sport-related head injury, suicide prevention

## Abstract

**Introduction:**

Sport-related head injury is increasingly recognised as a public health concern; however, research has predominantly focused on acute injury and individual recovery. Far less attention has been paid to how concussion and cumulative head injury are experienced and managed within families over time. In particular, the invisibility of concussion, the labour required of families to secure care, and the role of fragmented systems in shaping long-term harm remain underexplored. This study examines family experiences of living with and responding to the long-term consequences of sport-related head injury.

**Methods:**

A qualitative study was conducted using in-depth interviews with family members of individuals who had experienced sport-related head injury (*n* = 10). Participants included parents, partners, spouses and adult children. Data were analysed using reflexive thematic analysis following Braun and Clarke's approach. Analysis was iterative, inductive, and interpretive, focusing on patterned meaning across cases rather than prevalence.

**Results:**

Three interrelated themes were identified: (1) Struggling for recognition of an invisible injury, (2) Family advocacy as expected and exhausting labour, and (3) Fractured systems and preventing further harm. Families consistently described early identified recognition that something was wrong, which was repeatedly dismissed and/or minimised by medical, educational, and sporting institutions. The lack of recognition compelled parents and partners to assume prolonged advocacy roles, including care coordination, risk management, and crisis response, often in the absence of guidance or support. Health, education, and sport systems were experienced as siloed and reactive, with intervention frequently triggered only by crisis rather than functional impairment.

**Conclusions:**

Family experiences of sport-related head injury are shaped by the interaction between invisible injury, unrecognised expertise, and fragmented systems. Long-term harm is socially and institutionally produced through delayed recognition and the routine transfer of responsibility onto families, highlighting the need to reconceptualise concussion as a condition with enduring family-wide consequences.

## 1 Introduction

Sport-related head injury, most commonly conceptualised as a mild traumatic brain injury (mTBI), is a frequent occurrence in contact and collision sports and is increasingly recognised as a significant public health concern (1). Concussion is often understood to be a form of mTBI and is typically defined as a biomechanically induced brain injury resulting in transient neurological dysfunction, with symptoms that may include headache, dizziness, cognitive impairment, sleep disturbance, and emotional dysregulation (2). Although many individuals recover within weeks, a substantial minority experience persistent post-concussion symptoms that interfere with education, employment, and social participation (3).

The global incidence of concussion is high, with a large percentage of cases occurring annually through sport and recreation (4). Recovery trajectories are heterogeneous, and symptom resolution does not necessarily indicate neurological or functional recovery. Increasing evidence suggests that repetitive head impacts including both clinically diagnosed concussions and sub-concussive blows may be associated with cumulative neurological harm (5, 6). Reported long-term consequences include persistent cognitive impairment, mood disturbance, behavioural change, and, in epidemiological studies, it is speculated that neurodegenerative conditions such as Parkinson's disease, dementia, and chronic traumatic encephalopathy (CTE) are linked with repeated head impacts (7, 8). While causal pathways continue to be investigated, cumulative exposure to head impacts rather than concussion count alone appears to be a critical risk factor (9). Neurodegenerative outcomes associated with repetitive head injury may involve cognitive decline, personality and behavioural change, parkinsonism, and elevated risk of suicidality (10–12).

Despite growing biomedical evidence, there remains limited understanding of how long-term sport-related head injury is experienced and managed within families. Qualitative research suggests that families often encounter disbelief, fragmented care, and a lack of anticipatory guidance when symptoms are invisible, fluctuating, or delayed (13, 14). This can give rise to families feeling like they have been treated unfairly, whereby their experiential knowledge is discounted in favour of narrow clinical thresholds focused on more serious injury or structural imaging findings (e.g., magnetic resonance imaging (MRI) or functional magnetic resonance imaging (fMRI) (15, 16). As a result, parents and partners frequently assume extensive advocacy, care coordination, and risk-management roles without formal recognition or support (16).

Policy and practice responses to concussion have largely prioritised sideline assessment and graduated return-to-play protocols, with comparatively little attention to long-term follow-up, cumulative exposure, or family impact (17). Integration across health, education, and sport systems remains limited, and existing guidelines rarely address mental health sequelae, neurodegenerative risk, or the long-term educational and occupational consequences of injury (18–20). Understanding how families interpret, respond to, and live with long-term head injury is therefore critical to informing prevention strategies, care pathways, and policy reform (21).

This study attempts to provide a contribution towards understanding a family-centred, qualitative analysis of long-term outcomes following sport-related head injury across the life course. In addition, this research illustrates how mTBI is experienced, interpreted, and managed within families over time, including trajectories involving persistent post-concussion symptoms, epilepsy, suicidality and neurodegenerative disease. By integrating accounts from parents, partners, and adult children, the study shows the relational, cumulative, and systemic dimensions of harm.

Using reflexive thematic analysis (22–25), this study aims to extend current mTBI research by identifying inequalities, unpaid advocacy labour, and fractured institutional responses as central mechanisms shaping long-term outcomes. These findings attempt to understand mTBI as a public health issue with enduring family and societal consequences, and try to provide empirically grounded insights to inform policy and possible service redesign.

## 2 Methods

### 2.1 Study design and epistemological position

This qualitative study used a reflexive thematic analysis (TA) following Braun and Clarke's approach (22–25). A contextualist epistemological position informed the analysis, recognising that meanings are produced through the interaction between lived experience and wider social, institutional, and cultural contexts. This approach was deemed to be suitable for examining how families experience sport-related head injury, such as mTBI, and how these experiences are shaped over time by healthcare, education, and sports organisations.

### 2.2 Participants and recruitment

Participants were family members of individuals who had experienced sport-related concussion or reported cumulative head injury, oftentimes with associated long-term consequences. Eligible participants who were considered “family”, included parents, partners, spouses, and adult children who had been closely involved in care, advocacy, or daily support. Ten participants were recruited, representing ten individual family experiences.

The individuals who experienced head injury were all male, with an average age of 25 years (age range: 15–70 years). This wide age range may reflect the life-course nature of sport-related head injury, encompassing youth sport, elite and recreational adult sport, and later-life neurodegenerative outcomes (Table 1).

**Table 1:** Demographic characteristics of individuals represented in the study (*n* = 10).

Characteristic	Description
Sex	Male (*n* = 10)
Mean age at injury	25 years
Age range at injury	15–70 years
Relationship of participant to injured person	Parent, partner/spouse, adult child
Recruitment pathway	Specialist concussion clinic (*n* = 7); personal associations with research team (*n* = 3)
Injury context	Sport-related concussion and/or cumulative head injury
Long-term outcomes represented	Persistent post-concussion symptoms, epilepsy, suicidality, Parkinson's dementia, chronic traumatic encephalopathy, fatal traumatic brain injury
Time elapsed since the initial injury and/or symptom onset	>10 years to <2 years

Recruitment occurred via two pathways. Seven participants were recruited through a clinician working within a specialist concussion treatment clinic environment. These clinics specialise in the assessment and management of complex or prolonged concussion, meaning participants recruited through this route typically had experience of persistent symptoms, and/or delayed recovery, or reported effects of cumulative injury.

The clinician, identified individuals who met inclusion criteria (i.e., individuals who had experienced a sport-related concussion and/or had chronic symptoms) and enabled the provision of study information to this cohort. Interested participants were contacted by a member of the research team (ED) directly, providing additional information, ensuring voluntary participation and securing informed consent for the study.

The remaining three participants were recruited through personal associations with the research team (ED, LR), including prior professional and/or community contacts. These participants met the same inclusion criteria and were included to broaden the range of experiences beyond specialist referral pathways. No participants were currently under the direct care of any member of the research team. All participants provided informed consent prior to participation. Ethical approval was obtained from the research ethics committee of the Atlantic Technological University (reference number REC-ATUDG-25-0091).

### 2.3 Data collection

In-depth, semi-structured interviews were conducted with all participants via Microsoft Teams (Microsoft, WA, USA) with an average duration of fifty-seven minutes per interview. Interviews explored injury recognition, interactions with healthcare, education and sport systems, family impact, advocacy roles, and long-term consequences. Interviews were transcribed verbatim with identifying details removed to protect anonymity. Each transcript was cross checked with the original audio file (ED) and quality checked for accuracy by a research team member (LR).

### 2.4 Data analysis

Analysis followed Braun and Clarke's six-phase reflexive TA process (22–25): familiarisation, coding, theme development, review, definition, and writing. Coding was inductive, recursive, and reflexive, with themes conceptualised as patterns of shared meaning rather than categories or frequencies. Analysis focused on meaning across cases rather than comparison between individuals (Table 2).

**Table 2:** Theme, subtheme and descriptions.

Theme	Subtheme	Description
Theme 1: Struggling for recognition of an invisible injury	Knowing something is wrong, but not being believed	Families identified early changes following head injury, yet their concerns were frequently dismissed by professionals, leading to uncertainty and distress.
	Internalised doubt in the absence of validation and a desire for early, credible validation	Repeated dismissal led families to question their own interpretations of symptoms, producing self-doubt and self-gaslighting. Participants emphasised the importance of timely recognition and authoritative confirmation to legitimise concerns and guide care.
	Living with an invisible injury	Concussion-related difficulties lacked visible markers, rendering symptoms vulnerable to disbelief and minimisation.
	The emotional labour of constant explanation	Families undertook ongoing emotional work to explain, justify, and defend the reality of injury to others.
Theme 2: Family advocacy as expected and exhausting labour	Becoming an “expert by necessity”	In the absence of coordinated pathways, families acquired extensive medical and systems knowledge to support and protect their injured relative.
	Navigating fragmented and informal care pathways	Care was described as inconsistent and dependent on persistence, informal networks, or financial resources.
	Advocacy fatigue and emotional depletion	Prolonged advocacy resulted in exhaustion, burnout, and emotional depletion over time.
	Family life under chronic strain	Advocacy demands reshaped family routines, relationships, and emotional climates.
Theme 3: Fractured systems and preventing further harm	Health care thresholds focused only on catastrophic injury	Systems responded primarily to acute or life-threatening events, leaving persistent impairment unsupported.
	Prioritising liability over care	Institutions were perceived to prioritise procedural compliance and risk management over rehabilitation and support.
	Lack of concussion literacy among educators, coaches and employers	Limited understanding of concussion contributed to premature return-to-learn, work, or sport and increased harm.
	The inequity of access to specialist expertise	Access to knowledgeable clinicians and services was uneven and influenced by geography, resources, and networks.

Themes were identified inductively through sustained engagement with the dataset, while being sensitised by existing literature on concussion, traumatic brain injury, and family caregiving. Subthemes were used to add analytic depth rather than fragment the data, with overlap understood as analytically productive given the crossover of injury, family life, and systems. Participant identifiers (P1–P10) are used to support interpretive coherence rather than case-by-case reporting.

During manuscript development, initial themes were refined and integrated into higher-order themes to enhance analytic coherence and care by the research team (ED,LR). This process aligns with reflexive TA's emphasis on flexibility, reflexivity, and theoretical contribution over thematic quantity. Discrepancies in coding and theme development were resolved through dialogue between the coders, involving comparison of coded data extracts, review of themes, and refinement of thematic definitions. Divergent interpretations were revisited in relation to the original dataset with consensus reached through discussion to enhance interpretive coherence and transparency.

### 2.5 Reflexivity statement

The research team (ED and LR) adopted a reflexive qualitative stance, acknowledging that knowledge is co-constructed between participants and researchers. The authors have professional and research backgrounds in mTBI, sport, and health systems, which sensitised the analysis to issues of power, validation, and systemic failure. Rather than seeking analytic neutrality, reflexivity was embedded throughout coding, theme development, and interpretation, with ongoing attention to how researchers' assumptions shaped meaning-making.

### 2.6 Data trustworthiness

Trustworthiness was established adhering to qualitative research standards and reflexive thematic analysis. Credibility was supported by in-depth interviews and prolonged engagement with the data through repeated reading and reflexive memo-writing. Dependability and confirmability were enhanced through transparent documentation of analytic decisions, reflexive journaling, and regular research team discussion (ED,LR). Transferability was supported through description of participant contexts and injury trajectories. Ethical rigour was ensured through informed consent, confidentiality, and sensitivity to the emotional nature of the topic.

## 3 Results

This study explored family experiences (*n* = 10)of long-term concussion using reflexive thematic analysis. Three interrelated themes were identified (Table 3), illustrating how concussion operates as an invisible, family-wide trauma characterised by uncertain outcomes, systemic fragmentation, and enduring psychosocial impact.

**Table 3:** Policy and practice implications.

Themes and subthemes	Policy Implications	Practice Implications
Struggling for recognition of an invisible injury	Formal recognition of parental experiential knowledge	Training clinicians to validate uncertainty
Family advocacy as expected and exhausting labour	Public concussion awareness campaigns	Standardised concussion education materials
Fractured systems and preventing further harm	Clear, integrated concussion pathways	Case coordination support

### 3.1 Theme 1: struggling for recognition of an invisible injury

Across participant interviews, parents (6), adult children (1), spouses (2) and partners (1) consistently described an early recognition that something had profoundly altered following concussion or repeated head injury (Table 4). These concerns emerged long before diagnosis was acknowledged within formal medical or institutional systems. This recognition was frequently articulated through relational language, children or family members being described as “not himself” or fundamentally changed reflecting a disruption to identity rather than the presence of isolated symptoms. Despite this early awareness, participants reported repeated dismissal, minimisation, or contradiction by professionals, creating a sustained tension between the experiential knowledge of families and institutional authority.

**Table 4:** Case characteristics of head-injured individuals represented in the study (*n* = 10).

Participant (P)	Main sport	Sex	Relationship to participant
P1	Football	Male	Adult child
P2	Football	Male	Spouse
P3	Cricket	Male	Partner
P4	Rugby	Male	Parent
P5	Rugby	Male	Parent
P6	Rugby	Male	Spouse
P7	Rugby	Male	Parent
P8	Rugby	Male	Parent
P9	Football	Male	Parent
P10	Rugby	Male	Parent

The interviews highlighted the gap between lived observation and professional frameworks of concussion recovery. One participant (adult child) described knowing “*there was something really not right”,* yet repeatedly feeling unheard by clinicians, leading to self-doubt and emotional distress: “*It made me feel like I was completely bonkers”* (P1). This experience reflects the credibility of parental knowledge is systematically undermined, despite being uniquely positioned as close family members to observe subtle cognitive, emotional, and behavioural changes over time. Rather than reducing uncertainty, professional encounters often intensified it through inconsistent and shifting recovery timelines. As one participant noted, guidance moved arbitrarily from “three days” to “two weeks” to “six weeks”, depending not on clinical progression but on individual professional opinion (P1). Such contradictions eroded trust in health systems and left families without a coherent framework for understanding the evident ongoing difficulties.

The emotional impact of delayed validation was substantial. When concerns were eventually acknowledged, this moment was experienced as profound relief rather than reassurance, underscoring the cumulative toll of prolonged dismissal. Importantly, validation often came retrospectively, after minor or significant functional decline had already occurred.

Several narratives illustrate how this dismissal extended beyond uncertainty into active resistance to causal explanations. One participant (partner) describes prolonged frustration where concussion-related harm was never formally acknowledged as causative, even after the later development of epilepsy (P3). Clinicians' reluctance to recognise causal links contributed to a sense of injustice and left the family navigating complex neurological consequences without institutional support. Similarly, a parent suspected a greater concussion severity in their family member (son), this was followed by a delayed understanding of its neurological and cognitive implications, which suggested that even when injury is acknowledged, its long-term meaning is often minimised (P4).

Other parents described immediate recognition of catastrophic change, followed by sustained minimisation. For example P5 highlighted repeated partial reassurance and the absence of anticipatory guidance, leaving her unprepared for persistent difficulties (P5). Similarly P6 experiences were notable for alleged institutional dismissal and misattribution of symptoms to behaviour rather than brain injury, compounding stigma and delaying appropriate intervention, and knowing that “her husband” had fundamentally changed, long before a coherent medical explanation was available (P6).

In addition, P1 described that her father was “not right” long before medical recognition of neurodegenerative disease (P1). Similarly, a parent described early recognition paired with suspicion and disbelief from coaches and systems, highlighting how institutional cultures particularly in sport can actively contest family knowledge (P9). The evident failure to validate family knowledge contributes to delayed care, prolonged distress, and lasting mistrust, shaping the long-term impact of concussion not only on the injured individual, but on the family system as a whole.

#### 3.1.1 Knowing something is wrong, but not being believed

Across the interviews, participants described repeated experiences in which early concerns following concussion or repetitive head injury were dismissed, misdirected, or inadequately investigated by medical professionals. These specific encounters were characterised by narrow diagnostic framing, minimal assessment, and an emphasis on ruling out physical injury rather than recognising functional or evolving neurological impairment. Such responses contributed to prolonged uncertainty, delayed diagnosis, and escalating distress for both injured individuals and their families.

Several interviews illustrate how early symptoms were redirected away from neurology, delaying recognition of concussion-related harm. For example, P8 described how her initial concerns were channelled into cardiology rather than neurological assessment, resulting in reassurance rather than explanation: “*Initially my head went, oh, it's cardiology … and they said they weren't concerned”* (P1). Similarly, P3 described clinicians' reluctance to acknowledge a causal relationship between concussion and later epilepsy, despite accumulating evidence*: “The doctors haven't explicitly said that his epilepsy has come from his concussion… even though looking at the evidence … it would suggest that it is”* (P6). This lack of acknowledgement left family members feeling unheard and unsupported.

Emergency care settings were frequently described as sites of dismissal. One interviewee recounted a prolonged wait in casualty followed by minimal assessment and rapid discharge: “*We sat in casualty for four hours … he didn't even see a doctor … a junior doctor just gave us a leaflet and said read this”* (P4). For P4, this interaction conveyed that concussion was not regarded as a serious injury. Similarly, P5 described having to advocate forcefully for imaging despite clear neurological disturbance: “*We had to really force the A&E department to do that [schedule a CT scan]”* (P5). Discharge followed without meaningful guidance: “*They let him home … without any other guidance, anything at all”* (P5).

Dismissal was particularly harmful when neurological injury was reframed as behavioural or psychological choice. Participant 4 described how her sons symptoms were repeatedly invalidated: “*You sometimes felt they thought you were a hypochondriac … but you could see the symptoms”* (P4). This reframing coincided with escalating distress and suicidal ideation, with P4 observing, “*Every time the door was shut in your face, the angrier (her son) was becoming”* (P4). Despite loss of consciousness and severe injury, follow-up care was minimal: “*Basically we got MRI scans … nothing with doctors and stuff and no support with the school or anything”* (P4). Primary care further attributed symptoms to personal choice rather than injury: “*The doctor said, well, that's down to you. You need to make a choice whether to behave that way or not”* (P4).

Other participants described similar patterns of misattribution. Descriptions from P2 (spouse) regarding early observations of emotional and behavioural change were dismissed, with clinicians rejecting her credibility: “*we went to this consultant about his bladder and he more or less told me I was lying”* (P2). She went on to describe the early Parkinsonian features being misattributed to depression or cardiovascular issues “*We knew he wasn't right, we just assumed he had depression at that time because he had been away from home for a year on his own”* (P2), reflecting a missed occasion which may have delayed neurological recognition. P3 described her partners concussion as being poorly managed, with prolonged delay before specialist assessment: “*He'd obviously been struggling with it for quite a while … probably three to four weeks before anything was really put into motion”* (P3). Together, these accounts illustrate how professional dismissal may function as a structural barrier to timely, appropriate concussion care*.*

Participants described persistent uncertainty arising from shifting, contradictory, or absent medical timelines following concussion and/or repeated head injury. In some instances, even after specialist involvement, families reported an inability to obtain clear expectations regarding recovery, stabilisation, or prognosis. This ambiguity materialised as a central source of distress, shaping decision-making, trust in medical systems, and long-term family adaptation.

Several participants described repeatedly seeking clarity, only to be met with indefiniteness For example P2 articulated the ongoing uncertainty experienced during specialist care*: “Every time I'd ask … any idea how long this stage is going on for? And every time the answer was, no, not really”* (P2). Rather than reassurance, this absence of definitive timelines intensified anxiety, mirroring other accounts in which uncertainty itself became a significant burden. For P1, diagnosis and stabilisation unfolded over many years, with repeated reassessment rather than resolution: “*To get his medication right has taken probably about five or six years … and to get a diagnosis took about a year”* (P1). This prolonged trajectory reinforced uncertainty, offering no clear endpoint to recovery or adjustment.

In addition, timelines were frequently anchored to symptom disappearance rather than neurological recovery. P4 described a return-to-play decision based on rapid symptom resolution: “*He was not symptomatic within 48 h … so we kind of rushed him back”* (P4). Subsequent specialist assessment fundamentally reframed this understanding: “*(the specialist) … said he probably hadn't fully recovered from the first concussion”* (P4), highlighting the disconnect between early reassurance and later neurological interpretation. P5 similarly described initial expectations of rapid recovery “*We thought, oh, this is OK then, he'll wake up in the morning and be all right”* (P5) which were later overturned by specialist advice: “*He (neurological specialist) said it's at least three months from now”* (P5).

For P4, the absence of a coherent timeline was compounded by repeated system resets. “*Every time you had a medical alert, you'd go all the way back through the process”* (P4), preventing any sense of progression or closure. Within this context, her sons suicidal ideation and attempts cannot be understood as isolated events, but as emerging from prolonged brain injury, identity disruption, and systemic invalidation.

Diagnostic instability further destabilised meaning-making. P2 described her husband receiving multiple, incompatible diagnoses with shifting prognoses: “*The first diagnosis … he'd only probably last about three years … then it got downgraded to Parkinson's”* (P2). Rather than reassurance, these shifts forced repeated recalibration of expectations around decline and caregiving. P1 described timelines being retrospectively reconstructed following later diagnoses: “*We think it had started a long time before, but because he was still so very physically active, and even when his Parkinson's was very severe, he always exercised, that was just his thing”* (P1), forcing families to reinterpret years of symptoms without having had opportunities for timely intervention. P9 described fluctuating recovery expectations “*Some days he'd be like, ‘I think I feel a little bit better,’ and then other days … ‘I really don't feel right again'”* (P9) which undermined the ability to plan socially, professionally, or recover emotionally.

#### 3.1.2 Internalised doubt in the absence of validation

Participants frequently described internalising professional uncertainty and dismissal, leading to sustained self-doubt and what can be understood as “self-gaslighting”. Rather than simply reflecting a lack of information, this process involved the gradual erosion of confidence in their own perceptions, particularly when symptoms were invisible, fluctuating, or intermittently present. Across narratives, families described questioning the legitimacy of their observations even in the presence of persistent functional change.

P2s account illustrates how diagnostic ambiguity fostered ongoing self-questioning. She repeatedly interrogated whether observed changes were attributable to injury or personality: “*It's really hard to know how much that's been impacted by this and how much of that is just him”* (P2). This uncertainty reflects internalised doubt shaped by the absence of clear diagnostic confirmation. Similarly, P6 described questioning the validity of the causal link she perceived between concussion and later epilepsy: “*I think we always look for answers … but they just said there aren’t any”* (P6). Here, professional dismissal was absorbed into personal meaning-making, transforming uncertainty into self-blame or doubt rather than systemic critique.

P4s narrative demonstrates how cultural norms within sport encouraged minimisation and retrospective denial. “*It didn't seem that serious … it happens in rugby”* (P7). This opinion reflects not unawareness but internalised normalisation of concussion, with recognition of harm emerging only later. P5 similarly described how professional reassurance shaped her interpretation: “*We felt we would tell it wasn't a really serious brain injury … so we were relieved”* (P5). Yet this relief coexisted uneasily with instinctive certainty: “*Something instinctively (as a mother), I think, told me”* (P5), illustrating the tension between embodied knowing and external validation.

P6 described being positioned as overreactive, which contributed to questioning her own legitimacy as a mother and advocate: “*You sometimes felt that they felt you were a hypochondriac”* (P6). This repeated framing encouraged self-doubt despite observable decline. P2 described denial as both protective and imposed: “*I was a bit in denial … saying no, ‘it's all right’”* (P2). Analytically, this self-doubt functioned as a coping response to diagnostic ambiguity, delaying emotional and practical preparation for dementia and advanced care.

P1 reflected on how professional reassurance actively shaped family interpretations: “*We assumed it was all blood pressure related”* and described questioning the legitimacy of symptoms due to their inconsistency and invisibility: “*I also struggled at the start to kind of understand it because I'd look at him and be like, you look fine”* (P1). This illustrates self-doubt, produced by medical minimisation rather than naivety. Across the interviews, self-doubt emerged not as individual weakness, but as a structurally produced response to prolonged uncertainty.

A level of validation emerged as a pivotal emotional turning point, being “believed” did not function as a resolution of injury, but as a release from prolonged uncertainty, self-doubt, and isolation. Importantly, validation was often experienced as containment which provided direction, coherence, and legitimacy rather than reassurance or optimism about recovery.

For P4, finding a specialist marked a shift from navigating uncertainty alone to feeling guided within a credible clinical framework. “*Once I'd found (the specialist), then we were very much guided by him”* (P4). Here, validation did not imply recovery, but offered structure, objective expertise and a sense of leadership, enabling the family to orient themselves within a complex and uncertain trajectory. Similarly, P6 described validation emerging not through confirmation of causation, but through diagnostic recognition and ongoing monitoring: “*When he was diagnosed by a neurologist … that's when things at least had a name”* (P6).

Specialist assessment frequently produced a marked emotional shift by translating subjective concern into objective evidence. P4 described the moment of validation following formal testing*: “Then he did the Buffalo test … and it was like, oh my God, this really is having an impact on the brain”* (P4). Here, validation emerged through physiological measurement rather than reassurance, transforming doubt into a certain level of reassurance. P5 similarly described the profound impact of specialist consultation: “*Finding (a concussion specialist) … made a huge difference”* (P5). However, validation was emotionally complex; when expert opinion contradicted earlier reassurances, it triggered unhappiness rather than relief: “*When the expert said the opposite … that was really, really bad”* (P5).

For P4, meaningful validation did not come from public healthcare, but through third-sector support. “*It was only when we found a charity called Headway that really anything started to change”* (P4). This validation followed years of harm, underscoring systemic failures in recognising long-term brain injury. In neurodegenerative contexts, validation carried a distinct ambivalence. P1 described diagnosis as both relief and loss: “*We could deal with that …we were dealing with it. But then the dementia, like, cracks started to creep in, and then it got that (he) couldn't go out the house for about the last two years of his life” (*P1). This reflects a relief–grief dynamic, where legitimacy confirms irreversible decline. P2 similarly described diagnosis as clarity without comfort: “*That was a terrible light bulb moment for us (once the diagnosis of dementia was confirmed)”* (P2).

P9 described specialist diagnosis defined the injury as real and serious: “*Then there was obviously the realisation that they were like, yeah, you obviously have had (severe) concussion”* (P9). Participants expressed a strong desire for early, credible validation following concussion and neurological injury. Rather than seeking reassurance or cure, families described wanting timely recognition, informed investigation, and realistic expectation-setting. Early validation was viewed as protective mechanism capable of reducing distress, preventing harm, and supporting informed decision-making.

P2 described how identifying appropriate expertise transformed her experience: “*Finding the right person made a huge difference”* (P2). This validation did not alter the injury itself but provided coherence and direction. P6 similarly reflected on the missed opportunity for earlier investigation: “*Having more investigations and more conversations would have been helpful”* (P6). Here, early engagement was framed as a means of reducing uncertainty and validating parental concern. P5 highlighted the importance of early expectation-setting, noting, “*We would have been not so bad if we know what we're in for”* (P5). Uncertainty, rather than injury alone, was identified as a major source of distress.

For P4, the absence of early acknowledgment was central to later devastation. She stated, “*If they'd listened then, things might have been different”* (P4), explicitly linking delayed recognition to worsened outcomes, including suicidality. P10 critiqued superficial sideline assessments that substituted for meaningful recognition. He argued that pseudo-tests created false reassurance*: “Finger tests … Maddocks … you could answer all five correctly and still be suffering”* (P10). Early, credible validation grounded in neurological understanding rather than reassurance or minimisation. Participants positioned timely recognition as a critical intervention capable of altering trajectories and reducing family stressors.

#### 3.1.3 Living with an invisible injury

Participants described the invisibility of concussion and subsequent neurological injury as a central factor shaping their experiences of care, understanding of the injury, and the social response. Unlike visible physical injuries, concussion lacks external markers, rendering symptoms such as cognitive fatigue, nausea, emotional dysregulation, and behavioural change open to questioning and minimisation. Families repeatedly contrasted concussion with injuries that could be seen and therefore more readily believed. As one parent noted, “*If he'd broken his leg, you could see it. This, you can't see it, and people just don't get it”* (P5) and “*I honestly wished he'd broken his ankle. At least people would have understood”* (P5). These comparisons underscore how visibility functions as a representation of legitimacy within social and other contexts.

The absence of visible injury frequently led to social misunderstanding of symptoms as normative developmental or emotional issues. Parents described others attributing ongoing impairment to adolescence or mood rather than brain injury: “*People kept saying, ‘he's just a teenager.’ But being a teenager doesn't make you feel sick all the time”* (P8). Such minimisation obscured the seriousness of symptoms and delayed recognition of neurological harm. Concussion symptoms were described as subtle, fluctuating, and easily overlooked by institutions and peers, particularly when individuals “*appeared outwardly well”* (P8).

For several families, partial validation emerged only through formal diagnosis and monitoring. This validation was pragmatic rather than explanatory, legitimising symptoms without resolving uncertainty about cause or prognosis. However, for many, invisibility persisted even after diagnosis. For P4, the neurological injury was socially invisible, resulting in persistent misinterpretation of symptoms as behavioural or psychological, “*And a mental health team where everybody just kept repeating the process (repeated testing) all the time, and that's as much as I can say, the primary care was hopeless*” (P4).

Participants mentioned invisibility as a central burden shaping how concussion and neurological injury were interpreted, responded to, and managed. When injury lacked visible markers, impairment was frequently underestimated or dismissed by clinicians, peers, and wider social networks. Individuals often appeared “fine” to others, despite significant functional compromise. P9 described how her sons outward appearance masked impairment: “*From what she could see, he was fine … because he hadn't got anything broken or falling off him”* (P9). The absence of visible injury functioned as a barrier to recognition, placing the burden of proof on families.

This burden was particularly evident in conditions such as epilepsy and where post-concussion symptoms were evident or where impairment was intermittent. P3 described how her partners condition was only apparent during seizures, despite its permanence: “*Even now that it's under control, he still has a medical condition for life … just because he's on medication doesn't mean that he won't have another … (he's) still managing other lifestyle factors such as stress such as sleep”* (P3). Similarly, P4 described how his sons lack of overt symptoms contributed to minimisation: “*He hadn't got headaches … it just seemed like it wasn't that serious”* (P4). In contrast, the severity of impairment within the home could be profound. P5 described her sons functional dependence*: “He couldn't walk across the landing to the toilet without my husband being there to steady him … we had absolutely no idea that he'd be like that. You know, it was really scary”* (P5), a reality invisible to outsiders.

P4 described how her sons decline was observable within the family yet lacked external legitimacy: “*You could see him ageing … chest pains … flu-like symptoms”* (P4). Despite these signs, the absence of a visible injury led to ongoing misrecognition. In neurodegenerative contexts, invisibility was especially pronounced in early stages. P2 described her husband's emotional and cognitive changes: Neurodegeneration remains socially invisible until crisis points such as hallucinations or incontinence, delaying support and recognition “*There was no emotion … he just stood there”* (P2). P1 similarly described how physical fitness obscured decline: “*He was still very fit”* and with subtle signs “*He had no tremor … but he was really rigid”* going largely unnoticed to the general public.

In sport, invisibility directly shaped risk exposure. P9 described how her son appeared uninjured: “*You're not limping and you're not … those normal signs that you'd see for a sports person to be injured”* (P9). For P10, repeated returns to play following multiple impacts “*assessed for a minute and a half … and again allowed to play on”* (P10) demonstrate how symptoms were not treated as real unless dramatic. Invisibility transferred responsibility from systems to families, intensifying advocacy demands and intense emotional strain.

Participants frequently made explicit comparisons between concussion and injuries perceived as more “acceptable” or “legitimate”, particularly those involving visible structural damage. These comparisons shaped responses from families, peers, clinicians, and sports clubs, revealing a hierarchy of injury legitimacy in which visible musculoskeletal trauma is more readily understood and accommodated than brain injury. As one parent noted, “*He hadn't got anything broken … so I think he struggled to understand”* (P9), highlighting how the absence of visible damage impeded empathy and recognition.

Epilepsy and post-concussion conditions were similarly positioned as ambiguous and unsettling, eliciting uncertainty rather than sympathy. P6 described how invisibility complicated social understanding: “*People don't always understand it … it's not something you can see”* (P6). In acute care contexts, visible or catastrophic injuries often eclipsed concern for concussion. P7 described initial focus on a suspected neck fracture: “*They thought he'd broken his neck … hearing the consultant to say his neck was fine was almost relief”* (P7). Only later did concussion emerge as the more serious and enduring issue, illustrating how immediate, visible threats can dominate clinical attention.

P5 contrasted concussion with injuries which receive clearer validation, describing how absence of visible damage was equated with recovery: “*They said there wasn't a bleed or anything … so that means you're OK (with reference to her son)”* (P5). Similarly, P8 stated how her sons injury lacked recognition due to the absence of structural findings: “*There was no recognition for the fact that he had a severe brain injury”* (P8). P7 reflected on how normal imaging falsely reassured clinicians and families: “*They said there wasn't this … so we were relieved (after an MRI)”* (P7). In this instance, it may be understood that imaging-based reassurance reinforces biomedical hierarchies of legitimacy while obscuring functional impairment and/or progressive neurological decline (P7).

In amateur or professional sporting contexts, comparisons with “acceptable” injuries were particularly stark. P2 related how repeated physical contact and head impacts were normalised during her husband's football career: “*He didn't go to hospital or anything like that (if he felt concussed)”* (P2). Treating acute injuries as discrete events may have obscured cumulative neurotrauma and its long-term degenerative consequences. P9 explicitly contrasted concussion with visible injury: “*It's not a physical injury you can see, like a broken arm”* (P9). P10 further articulated this hierarchy, noting that while ankle injuries are treated as finite and non-existential, “*putting your brain on the line … it's a different matter”* (P10). Together, these comparisons reveal how cultural and clinical frameworks privilege visible injuries, systematically marginalising brain injuries despite its potentially life-altering consequences.

Beyond clinical settings, families encountered misunderstanding from peers, educational institutions, and sports clubs, where impairment was misinterpreted. This minimisation often redirected attention away from wellbeing and functional loss toward productivity and performance. P5 explained how injury discourse became centred on academic outcomes rather than health: “*Everybody wants to talk about … how's he getting on at school?”* (P5). Such comments implicitly seem to prioritise achievement over recovery, and not managing cognitive and physical strain.

Friends frequently underestimated the impact of fatigue, sensory sensitivity, and the need for lifestyle restriction. P6 stated how social invitations failed to account for post-exertional symptom exacerbation: “*They'll (friends) ask him to come out (socially) … and they don't quite understand the impact that can have on his exhaustion levels”* (P6). In educational contexts, misunderstanding was intensified by environmental demands. P4 described how the circumstances at school amplified sensory overload: “*In the evenings it can be quite noisy … and he just found that so overwhelming”* (P4). These environments rendered invisible symptoms such as fatigue and noise sensitivity socially obscured.

Several participants described how prolonged impairment was met with scepticism or dismissal, leading to social withdrawal. Helen noted, “*People just didn't get it … they thought we were making a fuss”* (P5). Rather than continually justifying their experiences, families often withdrew, further isolating both the injured individual and caregivers. In schools, behavioural interpretations frequently replaced neurological understanding. P4 described how the impairments were reframed as non-compliance: “*It was like, he's not cooperating … he's not being this, he's not being that (cooperating in a behavioural sense)”* (P4). This positioned the injured person as wilfully difficult rather than having impaired mental health.

P2 discussed a systemic erasure in the context of neurodegenerative illness: “*We fell through the cracks”* (P2). Dementia that does not align with age-related or stereotypical presentations particularly when sport-related is institutionally minimised. Similarly P2 described how early cognitive and behavioural changes were attributed to mood swings or ageing: “*We put that down to depression”* (P2), rendering Parkinson's dementia socially illegible until severe decline emerged.

In sporting contexts, disbelief was explicit. P9 explained how coaches questioned symptom legitimacy: “*Some coaches were like, ‘Is he making this up? Is this real?’”* (P9). P10 further critiqued the language of sport which trivialises brain injury: “*Sport's very good at playing down concussion … ‘a wee knock’, ‘a mild concussion’, ‘a slight knock’”* (P10). Collectively, these accounts demonstrate how social minimisation functions as a structural force, shaping delayed recognition and isolation.

#### 3.1.4 The emotional effort of constant explanation

Participants described the ongoing requirement to explain, justify, and translate concussion and neurological injury to others as a significant and exhausting form of emotional effort. Because symptoms were invisible, fluctuating, and poorly understood, families were repeatedly positioned as intermediaries between the injured person and social, educational, and healthcare systems. These efforts extended beyond information-sharing to include emotional containment, advocacy, and protection.

P5 described the cumulative emotional cost of repeated explanations: “*Every time you see a member of the friend or family, they ask how (her son) is”* (P5). While often well-intentioned, these interactions required continual emotional engagement of an unresolved situation. P3 similarly described becoming a gatekeeper of knowledge and safety, restricting information to manage risk and misunderstanding: “*I was one of the select few that knew about it”* (P3). This selective disclosure reflects the burden of deciding who can be trusted to understand and respond appropriately.

In educational contexts, explanation became an ongoing negotiation. P8 described repeated discussions with school staff to secure accommodations: “*There was lots of bringing him home from school early, giving him time, peace and quiet (due to persistent symptoms)” (P8)*. This advocacy required sustained effort and vigilance, reinforcing parental responsibility for managing institutional responses. Over time, the emotional cost of repeated questioning led some families to withdraw. P5 stated that her family were 'shutting up shop' as a coping strategy, noting*, “They stopped asking how he was, and we stopped saying”* (P5). Silence here functioned as protection against emotional depletion rather than resolution.

P4 described being forced into a defensive role, continually justifying her son's needs and behaviour: “*All you were doing was defending … why are you putting up with this behaviour?”* (P4). This perpetual defence positioned her as adversarial rather than supported. In neurodegenerative contexts, emotional labour intensified as decline progressed. P2 described becoming the sole translator of her husband's condition*: “It was just me and him … and I just dealt with things”* (P2). This ongoing effort encompassed both clinical interpretation and emotional containment, increasing as cognitive capacity diminished.

P8 similarly described the difficulty of navigating fragmented healthcare systems: “*Trying to navigate the healthcare system as a relative … that was so difficult”* (P8). Families functioned as interpretive advocates, making invisible neurodegeneration legible across systems. P3 elucidated similar demands in explaining her partners absence and performance: “*It's often me who answers the questions”* (P3).

### 3.2 Theme 2: family advocacy as expected and exhausting labour

Participants consistently stated being compelled into intensive advocacy roles, often in the absence of clear care pathways or institutional support. Rather than being guided by professionals, parents and partners were required to acquire expertise retrospectively, frequently after harm had already occurred. One parent captured this starkly: “*You’re an expert after the experience after you've made all your mistakes”* (P9). This learning-through-crisis positioned families as primary coordinators of care rather than supported recipients.

Care pathways were described as fragmented and heavily reliant on informal networks. Access to appropriate expertise often occurred by chance rather than design: “*It was only because of a friend of a friend that we even knew who to contact”* (P5). Such reliance on social capital created inequities in access and intensified the sense that families were navigating alone. The cumulative impact of this responsibility was described as deeply exhausting. One parent reflected, “*I was at the end of my tether. I was exhausted emotionally and physically”* (P8), underscoring the toll of sustained advocacy without respite.

Across the interviews, advocacy encompassed far more than appointment scheduling. This intensification of responsibility amplified advocacy fatigue and moral burden, particularly as decisions carried perceived serious consequences. These accounts depict parental and partner advocacy not as optional involvement, but as continuous labour structurally produced by fragmented systems of concussion and neurological care.

#### 3.2.1 Becoming an “expert by necessity”

Participants described a process of acquiring extensive medical, educational, and systems knowledge out of necessity rather than choice. In the absence of clear guidance, families were compelled to become “experts” in concussion, epilepsy, and neurodegenerative illness to protect their children, partners and parents. This expertise was not proactively provided, but developed reactively through experience, crisis, and repeated system navigation.

Several participants described undertaking independent research to compensate for gaps in professional support. P5 described investing significant time in identifying appropriate care*: “I spent a lot of time looking … I felt like I did a huge amount of research at that stage”* (P5). For P6, learning about epilepsy and concussion became essential to daily safety and long-term planning: “*I'm trying to learn as much about it as we can”* (P6). This learning was deemed as necessary for survival rather than empowerment.

P8 described rapidly acquiring concussion knowledge to advocate effectively for his son. Rather than relying on reassurance, he deferred to objective testing: “*I kept my mouth shut and just let the tests speak … then it was like, right, this is cognitive testing”* (P8). P5 similarly described how understanding emerged retrospectively, shaped by experience rather than instruction: “*We had absolutely no idea that he'd be like that”* (P5). Knowledge was built after deterioration occurred, limiting opportunities for preventative care.

P4 described knowledge acquisition as a process of repeated trial and rejection within fragmented systems: “*You just went back door after door trying to get what you needed … Just every time you banged the door, it was just been shut in your face”* (P4). This iterative process positioned families as system navigators without clear guidance. In neurodegenerative contexts, expertise was built through crisis rather than planning. P7 described responding reactively to her spouse's needs: “*If you needed something, we went and got it”* (P2). This expertise emerged in response to acute events such as hallucinations, bladder dysfunction, and paranoia, rather than through any form of guidance.

P1 highlighted how prior professional knowledge functioned as a protective factor: “*Because of my job, I knew the system a bit”* (P1). This underscores inequity for families without insider knowledge. These comments illustrate how families become “experts by necessity,” and absorb responsibility for knowledge production.

#### 3.2.2 Navigating fragmented and informal care pathways

Participants consistently described concussion and neurological care pathways as fragmented, informal, and highly dependent on individual persistence rather than coordinated systems. Access to appropriate expertise was shaped by geography, personal capacity, and social capital, producing uneven and often delayed care trajectories. Families reported navigating an unstructured network of services with little guidance, frequently moving between sectors to secure timely assessment.

Geographic inequity emerged as a significant barrier. P5 described the absence of local specialist provision: “*There was nobody in (our region) that does paediatric neurology”* (P5). As a result, families were required to travel or seek alternative routes to care at their own expense. P3 described moving between private and public systems to manage delays: “*We went private just to be seen quicker … then back under (public health) provision”*. This oscillation underlines how access to care was contingent on financial resources and the ability to navigate multiple systems simultaneously.

Several participants described access to specialist care as dependent on informal networks rather than formal referral pathways. P4 noted that without advocacy, families risked becoming trapped in cycles of delay: “*You could have been stuck in the system going around in circles”* (P4). P5 similarly described bypassing formal routes by mobilising personal contacts: “*I just went to friends and said … is there anyone (her son) could see?”* (P5). These accounts highlight how care was secured through improvisation rather than design.

P6 described persistence as the primary determinant of progress: “*You just weren't getting anywhere with absolutely nowhere”* (P6). Access to meaningful support similarly relied on informal connections. P2 described how assistance came through family networks rather than healthcare systems: “*(access to the specialist) was through (her son in law) … they trained together”* (P2). P1 echoed this reliance on personal capital: “*I had to pull on so many favours”* (P8).

Care fragmentation was further compounded in sporting contexts, particularly across jurisdictions. P9 described how protocols varied widely: “*Protocols are obviously different wherever you are (internationally)”* (P3).

#### 3.2.3 Advocacy fatigue and emotional depletion

Participants described advocacy as a cumulative and depleting form of persistent effort, with emotional fatigue intensifying over time rather than resolving. The sustained responsibility for monitoring risk, managing crises, and compensating for fragmented systems produced profound exhaustion, often layered onto existing caregiving, employment, and family responsibilities. P2 explicitly acknowledged the overwhelming nature of this burden: “*It was overwhelming for me alone, let alone for anyone else”* (P2).

The emotional toll of repeated crises was a recurrent feature across narratives. P6 described advocacy as “*extremely challenging … very exhausting and upsetting”*, reflecting the constant vigilance required to manage epilepsy and post-concussion risk. P4 similarly described the cumulative disturbance of prolonged uncertainty: “*The whole thing was quite disturbing and upsetting … it's your son”* (P4). P5 described the early stages as acutely overwhelming: “*It was really scary … quite tough right off”* (P5), noting that sustained worry was layered onto full-time employment and family life.

For P4, advocacy fatigue was inseparable from crisis containment. Her role extended into continuous suicide prevention, often without professional support: “*If it meant sitting in hospitals and refusing to move because I wanted to have a son at the end of it, then I wasn't moving”* (P4). Following a suicide attempt, she described physically transporting her son to safety: “*That resulted in me driving around London, getting him back home to get him safe.”* The cumulative emotional toll was enduring: “*It made you anxious permanently”* (P4). In this instance, advocacy functions as sustained crisis response rather than episodic support.

Exhaustion was compounded by the absence of a recovery horizon. P1 described reaching a breaking point: “*one of the times he got really, really poorly with the Parkinson's and I was at my wit's end”* (P1). Unlike concussion recovery, neurodegeneration offers no prospect of resolution, producing cumulative caregiver exhaustion and continued by saying “*I was sobbing with a therapist … about my dad (due to no prospect of recovery)”* (P8). Parkinson's dementia generates open-ended advocacy, extending emotional labour indefinitely.

P3 described the toll of isolation during acute management while travelling abroad: “*We spent about four days straight … curtains closed in a dark room”* (P3). Advocacy fatigue appears as a structurally produced outcome of prolonged uncertainty, inadequate support, and sustained responsibility for safety and care.

#### 3.2.4 Family life under chronic strain

The interviews reported an intense pressures (i.e., financial and moral) to act in the face of prolonged uncertainty and limited institutional support. The imperative to “do something” emerged not as an expression of choice, but as a response to perceived risk, responsibility, and fear of irreversible harm.

Several participants described private care as necessary rather than optional. P5 acknowledged the role of financial privilege in accessing care: “*We've been fortunate to have private healthcare … but not everybody may be in a position to do that … we're very lucky financially … if we hadn't been, we wouldn't be where we are now” (*P5), underlining structural inequities embedded in care access. P3 similarly described turning to private provision despite limited outcomes: “*We went private … but it wasn't overly helpful”* (P3). Nevertheless, private specialist care was framed as essential for legitimacy and reassurance. P8 described access to specialist expertise as pivotal: “*We're really lucky to see someone of the calibre of (the specialist they were referred to)”* (P8).

In contrast P4, financial burden was absorbed without meaningful alternatives: “*The rest of it (access to specialists), yeah, we've paid for”* (P4). This financial strain intersected with moral obligation, particularly in neurodegenerative contexts. P2 described rejecting institutional care as an ethical stance rather than a preference: “*There was no way we would have put him in a home*” (P2). Caregiving here becomes a moral obligation, amplifying burden and isolation. P2 similarly described care decisions as ethically charged and possibly under time constraints: “*How long is this money going to last?”* (P2). Neurodegeneration transforms care into an enduring moral responsibility, intensifying stress and guilt over resource allocation.

Financial and moral pressures intersected with career insecurity, for example P3 described the tension between protecting health and maintaining professional viability: “*You've got to do the right thing … but also don't want to take too much time (being unavailable after a head injury)”* (P93. The pressure to act financially, ethically, and professionally was structurally produced by fragmented systems, leaving families to bear the consequences of decisions made under constraint rather than genuine choice.

### 3.3 Theme 3: fractured systems and preventing further harm

Participants described an erosion of trust across healthcare, education, and sports organisations, shaped by repeated experiences of dismissal, inconsistency, and fragmentation. Rather than functioning as protective infrastructures, these systems were perceived as reactive and insufficiently attuned to the complexity of concussion and neurological injury. Within healthcare, responses were frequently described as narrowly focused on identifying physical injury while overlooking functional impairment. One parent noted, “*Unless he couldn't walk or speak, there was no interest. Fine functioning? Not interested”* (P8). This threshold-based approach undermined confidence in clinical care and left families managing significant impairment without support. Trust was further eroded by perceptions of institutional defensiveness in education settings. Schools were described as prioritising liability management over student wellbeing: “*They were scared we were going to sue them, that was obvious”* (P5). Such responses positioned families as adversaries rather than partners in care.

Outdated beliefs about concussion further contributed to mistrust. Educators and coaches were perceived as relying on simplistic recovery narratives, as illustrated by one account: “*The PE teacher said, ‘concussion only lasts two weeks.’ That was it”* (P7). Commonly sports, medical, and support systems failed to integrate responsibility, resulting in fragmented and contradictory guidance. These interviews demonstrate that fractured trust is not an attitudinal problem, but a rational response to systems that consistently fail to recognise, integrate, and respond to long-term neurological injury.

#### 3.3.1 Health care thresholds focused only on catastrophic injury

Participants depicted healthcare systems as operating on high thresholds for intervention, with care triggered primarily by physical or life-threatening injury rather than functional, cognitive, or psychosocial impairment. This threshold-based model produced reactive rather than preventative or rehabilitative care, leaving families to manage ongoing neurological harm without sustained support. Several participants described early warning signs being minimised as isolated or insignificant events. P6 recounted how initial seizures were dismissed: “*They said it was probably just one of those freak things”* (P6), delaying recognition of epilepsy as a chronic condition. In emergency settings, assessment was narrowly focused on ruling out acute pathology. P4 described how access to care depended on visible crisis: “*Unless you're bleeding or dying, you just don't get what you need”* (P4). P9 similarly described reassurance based solely on imaging: “*He's not got a bleed on the brain … so he doesn't need to be hospitalised”* (P9), despite significant functional impairment.

Once individuals survived the acute injury phase, ongoing support frequently disappeared. P4 described primary care as “hopeless,” noting that clinicians “*didn't recognise that injury”* (P4). In this context, her sons suicidality intensified following systemic abandonment. Despite repeated suicide attempts, responses remained episodic: “*They wouldn't keep him there (in the hospital or institution)”* (P4). Suicidality was contextualised as emerging after the head injury and exacerbated by care denial: “*They didn't recognise that injury … and the more doors were shut, the angrier he (her son) was becoming”* (P4).

In neurodegenerative cases, care was similarly minimal, shallow and administrative. P2 described support focused on diagnosis rather than relationship or continuity: “*They done the test (cognitive testing) on the phone”* (P2). Neurodegeneration was managed administratively rather than relationally, further undermining trust where some symptoms were dismissed outright: “*He (clinician) more or less told me I was lying”* (P2).

P9 articulated mistrust arising from inconsistent concussion governance, where early care relied on self-report rather than structured assessment: “*A lot of it was left up to him … asking how are you feeling today?”* (P9). P10 highlighted a boundary problem between sport and health systems, noting that severity was recognised only once injury entered emergency medicine*: “As soon as he entered the emergency department, it was traumatic brain injury (and not a sport related concussion) … like a car crash”* (P10).

#### 3.3.2 Prioritising liability over care

Many participants described educational and workplace systems as prioritising risk management and liability over sustained care and accommodation following concussion and neurological injury. While return-to-play protocols were often present, participants noted a striking absence of equivalent return-to-learn or return-to-work frameworks. P5 observed, “*Return to learn was never mentioned … that terminology hadn't been used at all*” (P5), highlighting a structural gap in educational responses to brain injury.

In both school and employment contexts, safety concerns frequently superseded rehabilitative support. P3 discussed workplace discussions framed around risk rather than recovery: “*There were conversations about whether he was safe to do his job (after an epileptic episode)”* (P6). Similarly, P7 described how school responses were driven by procedural compliance: “*Immobilising him and calling an ambulance (in the absence of a pitch side medic and without identifying a concussion) … that's school policy and risk assessment”* (P7). While such actions mitigated immediate liability, they did not translate into ongoing support or adaptation.

Early institutional responses often normalised absence or impairment, delaying recognition of deeper change. P8 described how initial school absence was treated as routine illness: “*They just accepted it as a sickness absence”* (P8). Only after the fact, when deterioration became more apparent in her son, did the school begin to acknowledge the extent of neurological impact. In other cases, behavioural framing replaced neurological understanding. P4 described how learning difficulties were interpreted as non-compliance: “*They just said he wasn't cooperating”*. This seemed to shift responsibility onto the injured individual rather than the institution.

In education settings, liability-driven approaches were compounded by limited medical expertise. P10 described how grassroots and school rugby relied on volunteers rather than trained clinicians: “*Grassroots and schools rugby … dependent on volunteers … you don't have qualified medics … coach was a linesman and the first aider”* (P10). These descriptions illustrate how prioritisation of liability over care leaves injured individuals insufficiently supported, reinforcing mistrust and exacerbating long-term harm.

#### 3.3.3 Lack of concussion literacy among educators, coaches and employers

Participants identified inadequate concussion literacy among educators and coaches as a key contributor to harm, delayed recovery, and fractured trust. Across school and sporting contexts, decisions about return-to-learn and return-to-play were frequently made without understanding the neurological vulnerability associated with concussion. Academic expectations were often resumed rapidly despite ongoing symptoms. P5 described how her son was expected to return to full academic participation within days*: “Within 48 h they were expecting him to be back in full lessons”* (P5). This absence of graded return-to-learn guidance placed students at risk of symptom exacerbation. In sporting contexts, similar patterns were evident. P6 described her husband being returned to play immediately following loss of consciousness: “*He was told to go straight back onto the pitch once he woke up”* (P6). P7 similarly described how his son continued playing despite amnesia: “*He played on for the whole first half … he doesn't remember any of it”* (P7), highlighting a failure to recognise covert symptoms.

Several participants described complete absence of anticipatory guidance. P5 noted, *“We had no knowledge … that he might be like that”* (P5), illustrating a systemic failure to educate families and schools about post-concussion trajectories. In some cases, immediate return-to-play practices directly contributed to harm. P4 described how her son was physically returned to the field: “*They picked him up and put him back on the pitch”* (P4).

P10 provided a detailed critique of sideline practices, describing superficial assessments mistaken for concussion screening: “*Coach runs on ‘are you OK to play on’ … thumbs up … coach does the finger test”* (P10). It is evident that a lack of concussion literacy existed among educators and coaches which may result in premature return, cumulative injury, and prolonged harm, reinforcing the need for mandatory training and integrated return-to-learn and return-to-play frameworks.

#### 3.3.4 The inequity of access to specialist expertise

Participants consistently described access to specialist concussion and neurological expertise as contingent, unpredictable, and poorly aligned with clinical need. Rather than being systematically offered, specialist care was frequently discovered by families through persistence, chance encounters, or informal networks. P2 articulated the early stages of this process as obstructive and disorienting: “*Working out who do I need to see … it just felt like brick walls”* (P2). This absence of clear referral pathways delayed appropriate assessment and intensified uncertainty.

For some families, specialist involvement occurred only after repeated deterioration. P6 described how epilepsy was diagnosed following recurrence rather than prevention: “*It was when he had his next one (epileptic episode) that he was then diagnosed”* (P6). This reactive approach highlights how escalation, rather than early recognition, functioned as the gateway to expertise. P8 similarly framed referral as contingent on circumstance rather than structured care: “*We were really lucky we were sent to the right person at the right time”* (P8). The language of “luck” underlines the absence of reliable, needs-based pathways.

Several participants described specialist care emerging through informal channels. P5 noted, “*It was* via *friends … and we contacted him”* (P5), illustrating how personal networks substituted for institutional coordination. Continuity of care was also described as fragile and vulnerable to disruption. P1 highlighted how professional insider knowledge functioned as a protective factor*: “That was definitely because I knew the system”* (P1). This emphasises inequities faced by families without healthcare literacy or system familiarity. For P3, meaningful specialist engagement occurred only after returning home: “*Then he went to see a specialist (due to the unavailability of a suitable specialist while travelling)”* (P3), suggesting that geography, rather than clinical urgency, governed access. The absence of systematised referral mechanisms leaves families dependent on chance, social capital, financial resources and personal endurance, contributing to delayed diagnosis, fractured care, and prolonged harm.

## 4 Discussion

This study sought to understand family experiences of long-term outcomes following sport-related head injury. The identified themes demonstrate that the consequences of long-term concussion is not simply a function of injury severity or recovery duration, but is multi-faceted. which ultimately transfers the majority of responsibility to families, in the absence of coordinated, preventative care pathways (26, 27).

### 4.1 Theoretical interpretation

Across cases, families' accounts were unified by persistent difficulty securing recognition of concussion as a legitimate, ongoing brain injury. Recognition failed not only at the point of injury, but repeatedly across time, even as symptoms persisted or escalated. This reflects a form of unfairness, whereby families' experiential knowledge was discounted in favour of medical frameworks oriented toward acute, observable pathology (28, 29). In concussion, where injury is often invisible and recovery non-linear, credibility became contingent on symptoms conforming to expected timelines, when they did not, families were left in prolonged states of uncertainty (30, 31).

Analysis of the data demonstrated how advocacy became structurally necessary. Families were not merely supporting recovery but compensating for systemic gaps coordinating care, monitoring risk, negotiating accommodations, and responding to crisis (32, 33). Importantly, advocacy was not framed as temporary; it persisted across educational transitions, employment decisions, and, in some cases, suicidality and neurodegenerative decline (34).

The often fractured systems and disconnect between health, education, and sport, required families to manage the consequences of disorganisation (35). Severe outcomes were therefore not experienced as random, but as the cumulative result of delayed recognition, transferred responsibility, and missed opportunities for early intervention. This challenges individualised risk narratives and reframes sport-related head injury as a systemic and public health issue (36).

### 4.2 Policy and practice implications

The findings of this study have direct implications for health, education, and sport policy in relation to families and long term care givers. Early validation of family concerns, clear expectation-setting, and structured follow-up pathways are essential to reduce prolonged uncertainty and prevent escalation into chronic impairment, suicidality, or neurodegenerative outcomes (37, 38).

These data demonstrate that **concussion literacy must extend beyond clinicians** to the wider network surrounding injured individuals, including educators, coaches, and employers. Literacy efforts should emphasise symptom variability, cumulative exposure, and the limitations of brief sideline assessments (39).

### 4.3 Impact and knowledge translation

This study contributes to knowledge translation by making visible the family-level consequences of long-term concussion. By focusing on family members (i.e., partners, parents, and siblings), the research translates private, often hidden stresses and efforts into empirically grounded knowledge which can inform clinical guidance, school policies, and sport governance (39, 40). Rather than treating concussion recovery as an individual biomedical process, the findings demonstrate the need for systems-based responses which reflect lived complexity.

### 4.4 Academic impact

While persistent post-concussion symptoms are well documented, less is known about how failures of recognition and fragmented care reshape family roles, relationships, and life trajectories over time. By examining long-term outcomes including suicidality and neurodegeneration, this research extends the discussion about the unseen effects of concussion beyond recovery models and contributes to understanding concussion as a chronic, socially mediated condition.

### 4.5 Strengths and limitations

A key strength of this study is its depth across ten cases spanning different sports, life stages, and outcomes. Reflexive thematic analysis enabled examination of patterned meanings rather than prevalence. Limitations include retrospective accounts and the absence of injured individuals' voices in all cases; however, family members are uniquely positioned to observe longitudinal change, cumulative impact, and system interaction.

## 5 Conclusion

This study demonstrates that long-term harm following sport-related head injury is not rare, accidental, or confined to elite sport. Instead, it reflects predictable outcomes of invisible injury, transferred responsibility, and fractured systems. Addressing these harms requires coordinated cultural, clinical, and policy change that recognises families not as peripheral advocates, but as central actors bearing the consequences of systemic failure.

## Data Availability

The raw data supporting the conclusions of this article will be made available by the authors based on reasonable requests.

## References

[1] McKee AC Mez J Abdolmohammadi B Butler M Huber BR Uretsky M. Neuropathologic and clinical findings in young contact sport athletes exposed to repetitive head impacts. JAMA Neurol. (2023) 80(10):1037–50. 10.1001/jamaneurol.2023.2907 37639244 PMC10463175

[2] Tierney G. Concussion biomechanics, head acceleration exposure and brain injury criteria in sport: a review. Sports Biomechanics. (2024) 23(11):1888–916. 10.1080/14763141.2021.2016929 34939531

[3] Heslot C Azouvi P Perdrieau V Granger A Lefèvre-Dognin C Cogné M. A systematic review of treatments of post-concussion symptoms. J Clin Med. (2022) 11(20):6224. 10.3390/jcm11206224 36294545 PMC9604759

[4] Peterson AB Waltzman D Daugherty J Chen J Breiding M. Sport and recreation related concussion in children: national concussion surveillance system. Am J Prev Med. (2024) 67(3):370–9. 10.1016/j.amepre.2024.05.003 38852098 PMC11338698

[5] Ntikas M Binkofski F Shah NJ Ietswaart M. Repeated sub-concussive impacts and the negative effects of contact sports on cognition and brain integrity. Int J Environ Res Public Health. (2022) 19(12):7098. 10.3390/ijerph19127098 35742344 PMC9222631

[6] Walter AE Wilkes JR Arnett PA Miller SJ Sebastianelli W Seidenberg P. The accumulation of subconcussive impacts on cognitive, imaging, and biomarker outcomes in child and college-aged athletes: a systematic review. Brain Imaging Behav. (2022) 16(1):503–17. 10.1007/s11682-021-00489-6 34308510

[7] Smolen P Dash PK Redell JB. Traumatic brain injury-associated epigenetic changes and the risk for neurodegenerative diseases. Front Neurosci. (2023) 17:1259405. 10.3389/fnins.2023.1259405 37795186 PMC10546067

[8] Murray HC Osterman C Bell P Vinnell L Curtis MA. Neuropathology in chronic traumatic encephalopathy: a systematic review of comparative post-mortem histology literature. Acta Neuropathol Commun. (2022) 10(1):108. 10.1186/s40478-022-01413-9 35933388 PMC9356428

[9] Morales JS Valenzuela PL Saco-Ledo G Castillo-Garcia A Carabias CS McCrory P. Mortality risk from neurodegenerative disease in sports associated with repetitive head impacts: preliminary findings from a systematic review and meta-analysis. Sports Med. (2022) 52(4):835–46. 10.1007/s40279-021-01580-0 34674184

[10] Kay JJ Coffman CA Harrison A Tavakoli AS Torres-McGehee TM Broglio SP. Concussion exposure and suicidal ideation, planning, and attempts among US high school students. J Athl Train. (2023) 58(9):751–8. 10.4085/1062-6050-0117.22 36252208 PMC11215744

[11] Iverson GL Gaudet CE Karr JE. Examining suicidality in adolescents who have sustained concussions. J Neurotrauma. (2023) 40(7-8):730–41. 10.1089/neu.2022.0233 36006376

[12] Fralick M Sy E Hassan A Burke MJ Mostofsky E Karsies T. Association of concussion with the risk of suicide: a systematic review and meta-analysis. JAMA Neurol. (2019) 76(2):144–51. 10.1001/jamaneurol.2018.3487 30419085 PMC6439954

[13] Chadwick L Marbil MG Madigan S Callahan BL Yeates KO. The relationship between parental and family functioning and post-concussive symptoms after pediatric mild traumatic brain injury: a scoping review. J Neurotrauma. (2024) 41(3-4):305–18. 10.1089/neu.2023.0201 37565282

[14] Quatman-Yates CC Miley AE Morrison P Hugentobler J Wade SL Rhine TD. Adolescent and parent perceptions of the impact of concussion/mTBI on family functioning and activity levels in recovery. J Head Trauma Rehabil. (2022) 37(4):E280–91. 10.1097/HTR.0000000000000725 34570028

[15] Nelson LD Gray S Barry CO Young SA deRoon-Cassini TA Hedayat H. Patient experiences of and priorities for traumatic brain injury health care in a US level 1 trauma center: a qualitative study. Mayo Clin Proc Innov Qual Outcomes. (2025) 9(4):100630. 10.1016/j.mayocpiqo.2025.100630 40584562 PMC12205785

[16] Jones TM Bhanji A Osman S Cai XC Garfinkel S Weinstein AA. Experiences of caregivers and individuals living with traumatic brain injury in accessing health information: a qualitative investigation. Brain Inj. (2023) 37(4):293–302. 10.1080/02699052.2022.2145366 36453445

[17] Davies SC Bernstein ER Daprano CM. A qualitative inquiry of social and emotional support for students with persistent concussion symptoms. J Educ Psychol Consult. (2020) 30(2):156–82. 10.1080/10474412.2019.1649598

[18] Drattell J Kroshus E Register-Mihalik J D’Lauro C Schmidt J. Barriers to delivering concussion education: identifying opportunities for change through the capability, opportunity, motivation, behavior (COM-B) model. Health Educ Behav. (2025) 52(2):190–8. 10.1177/10901981241292274 39462297

[19] Badenhorst M Skilton D Zoellner A Lucas P Salmon DM Walters S. Sport, healthcare and educational organisations’ perceptions of a framework for managing concussion in New Zealand schools: a qualitative study. J Prim Health Care. (2025). 10.1071/HC24190 41145143

[20] Seguin CM Culver DM. The lived experience of sport-related concussion: a collaborative inquiry in elite sport. J Appl Sport Psychol. (2022) 34(5):916–37. 10.1080/10413200.2021.1998804

[21] Kerr ZY Gildner P Parker SK Kostogiannes V Callahan CE Nedimyer AK. Sport culture and communication among middle school athletes, parents, and staff: a qualitative study. PLoS One. (2023) 18(3):e0282252. 10.1371/journal.pone.0282252 36920886 PMC10016647

[22] Braun V Clarke V. Toward good practice in thematic analysis: avoiding common problems and be (com) ing a knowing researcher. Int J Transgend Health. (2023) 24(1):1–6. 10.1080/26895269.2022.2129597 36713144 PMC9879167

[23] Braun V Clarke V Hayfield N. A starting point for your journey, not a map’: nikki hayfield in conversation with Virginia braun and Victoria clarke about thematic analysis. Qual Res Psychol. (2022) 19(2):424–45. 10.1080/14780887.2019.1670765

[24] Braun V Clarke V. Conceptual and design thinking for thematic analysis. Qual Psychol. (2022) 9(1):3. 10.1037/qup0000196

[25] Braun V Clarke V. Reporting guidelines for qualitative research: a values-based approach. Qual Res Psychol. (2025) 22(2):399–438. 10.1080/14780887.2024.2382244

[26] Muliira JK Lazarus ER Jacob D Roslin H. The needs of families caring for patients with traumatic brain injury: a scoping review. Disabil Rehabil. (2024) 46(20):4586–94. 10.1080/09638288.2023.2278178 37933167

[27] Matney C Bowman K Berwick D. National academies of sciences, engineering, and medicine. Rehabilitation and long-term care needs after traumatic brain injury. In: Berwick D, editor. InTraumatic Brain Injury: A Roadmap for Accelerating Progress. National Academies Press (US) (2022). 35533242

[28] Joghataie G Hundal S Mushtaque A Tator CH Tartaglia MC. Critical gap in practice—lack of attention to falls and possible fall-related post-concussion symptoms in older adults and individuals with neurodegenerative disease. GeroScience. (2025) 47(1):1269–76. 10.1007/s11357-024-01312-y 39207649 PMC11872947

[29] Paladino J Chavez Granados H Connor Eruchalu JA Davila C Ramirez Gomez L Sideman AB. A qualitative study to characterize the experiences of patients and caregivers with dementia diagnostic disclosure communication and care planning. J Geriatr Psychiatry Neurol. (2026) 39:08919887251388036. 10.1177/08919887251388036 PMC13181747 41092108

[30] Kim I. Adolescent concussion in high school Athletes: Neurodevelopmental Risks, Diagnostic Biomarkers, and Gaps in Clinical Management.

[31] Sheldrake E Al-Hakeem H Lam B Goldstein BI Wheeler AL Burke M. Mental health outcomes across the lifespan in individuals with persistent post-concussion symptoms: a scoping review. Front Neurol. (2022) 13:850590. 10.3389/fneur.2022.850590 35481264 PMC9035995

[32] Kroshus E Steiner MK Chrisman SP Lion KC Rivara F Lowry SJ. Improving post-concussion discharge education for families seeking emergency department care: intervention development. Brain Inj. (2024) 38(6):479–88. 10.1080/02699052.2024.2318595 38441083 PMC11283255

[33] Gomez D Glang A Haarbauer-Krupa J Bull R Tucker P Ratcliffe J. Stakeholder perspectives on navigating the pediatric concussion experience: exploring the needs for improved communication across the care continuum. NeuroRehabilitation. (2023) 52(4):605–12. 10.3233/NRE-220220 37125574 PMC10481243

[34] Aza A Gómez-Vela M Badia M Begoña Orgaz M González-Ortega E Vicario-Molina I. Listening to families with a person with neurodegenerative disease talk about their quality of life: integrating quantitative and qualitative approaches. Health Qual Life Outcomes. (2022) 20(1):76. 10.1186/s12955-022-01977-z 35525943 PMC9077340

[35] Karmali S Beaton MD Babul S. Outlining the invisible: experiences and perspectives regarding concussion recovery, return-to-work, and resource gaps. Int J Environ Res Public Health. (2022) 19(13):8204. 10.3390/ijerph19138204 35805862 PMC9266414

[36] López Frías FJ McNamee M. Concussion and brain injuries in sport: conceptual, ethical and legal perspectives. Sport Ethics and Philosophy. (2024) 18(3-4):259–66. 10.1080/17511321.2024.2370583

[37] Xiang L Bansal S Wu AY Roberts TL. Pathway of care for visual and vestibular rehabilitation after mild traumatic brain injury: a critical review. Brain Inj. (2022) 36(8):911–20. 10.1080/02699052.2022.2105399 35918848 PMC10134507

[38] Moore BM Stark RK D'Angelo EC. Multidisciplinary care for patients with persistent symptoms following concussion: a systematic review. Disabil Rehabil. (2024) 46(9):1760–75. 10.1080/09638288.2023.2205663 37147858

[39] Salmon DM Badenhorst M Keung S Kerr ZY Register-Mihalik JK Romanchuk J. Utilisation of New Zealand rugby’s concussion management pathway: a mixed methods investigation. Eur J Sport Sci. (2024) 24(12):1883–902. 10.1002/ejsc.12213 39500620 PMC11621389

[40] Tucker PM Strizak J Rieger B Lounsbury S Leddy J. The unconsidered pathway: suggestions for physical therapists to facilitate student reintegration to physical education after a concussion. Children. (2024) 11(10):1206. 10.3390/children11101206 39457171 PMC11506483

